# Dying Depositions

**Published:** 1901-06-01

**Authors:** 


### DYING DEPOSITIONS.

At the Central Criminal Court on May 17, according to the report in the *Times*, Mr. Sydney Smith, 34, surgeon, was indicted for and charged on the coroner's inquisition with the wilful murder of Mrs. Florence Madeline Bromley Smith, and he was also indicted for using an instrument or means unknown with intent to procure her miscarriage. He was found *Not Guilty* on both counts and we cannot but feel that he was very hardly used in being charged at all in view of the evidence which was offered. A point of interest in the trial was that much, everything in fact, hinged upon a statement made by the deceased. This statement, it was contended, was not admissible, because it had not been shown that when it was made the deceased was in the expectation of immediate death, and also on another ground which one much less frequently hears brought forward, namely, that it was "made in answer to questions." Mr. Justice Bruce said that he thought the statement could not be admitted. It was very necessary in these cases to take great care that these declarations were not admitted unless they came strictly within the rules. In order to make the statement admissible it must clearly appear that the person making it had abandoned all hope of recovery. Here that did not appear. There was the further point that the statement was made in answer to questions; and, following the ruling of Mr. Justice Cave, he thought the statement was not admissible.

